# Trait Plasticity and Warming Vulnerability in a Structurally Diverse Seagrass Ecosystem

**DOI:** 10.1002/ece3.72011

**Published:** 2025-08-21

**Authors:** Cloverley M. Lawrence

**Affiliations:** ^1^ Scientific Services South African National Parks Sedgefield South Africa; ^2^ Institute for Coastal and Marine Research Nelson Mandela University Port Elizabeth South Africa

**Keywords:** acclimation, climate change, GAMMs, phenotypic plasticity, seagrass resilience, *Zostera capensis*

## Abstract

Seagrass ecosystems deliver critical ecological functions but are increasingly threatened by climate change and local stressors. In temperate lagoons, thermal stress, turbidity and tidal exposure influence the structure and persistence of seagrass meadows. We investigated spatial and seasonal variability in *Zostera capensis* morphology and density in Langebaan Lagoon, South Africa. Field surveys and environmental monitoring revealed strong spatial contrasts: shallow, exposed sites supported small‐leaved morphotypes with high shoot densities but low biomass, while deeper, cooler sites harboured sparse large‐leaved morphotypes with greater canopy height and biomass. Seasonal diebacks were pronounced in late summer, coinciding with elevated temperature and irradiance, followed by regrowth in autumn. Generalised additive mixed models identified temperature, turbidity and tidal exposure as the strongest predictors of seagrass density, jointly explaining over 80% of variation. Experimental investigations confirmed thermal thresholds, with both morphotypes declining at > 26°C, indicating vulnerability to future warming. Morphological plasticity enabled some acclimation, but small‐leaved morphotypes did not outperform large‐leaved morphotypes under heat stress. Epiphyte loads and faunal associations were greater in large‐leaved beds, suggesting potential trophic consequences if these morphotypes decline. Historical imagery indicates long‐term *Zostera* contraction near the lagoon mouth, where large‐leaved morphotypes dominate, raising concerns about habitat simplification. With ongoing climate warming, large‐leaved morphotypes may persist in the short term, but further loss of structurally complex beds is likely, with cascading effects on biodiversity and ecosystem function. Conservation efforts should prioritise mitigating local stressors and preserving thermal refugia to support seagrass resilience.

## Graphical abstract

*Zostera capensis* in Langebaan Lagoon exhibits marked spatial and seasonal variation in morphology, driven by temperature, turbidity and tidal exposure. Field results show that small‐ and large‐leaved morphotypes reflect phenotypic plasticity in response to environmental gradients, with both forms approaching their upper thermal limits under experimental scenarios. These findings highlight site‐specific vulnerability and underscore the need for locally tailored seagrass conservation strategies under climate change.

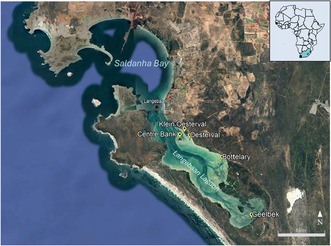

## 1 Introduction

A central aim in ecology and conservation remains the contribution to understanding of environmental and biological drivers shaping species distribution, abundance and demography, across geographical ranges (Parmesan 2006; Burgess et al. 2020). Species‐level differences in traits such as morphology and population density can reflect both evolutionary history and local environmental conditions, often underpinned by patterns in genetic diversity (Oliva et al. 2014; Mascaró et al. 2014). Seagrass ecosystems, found in nearshore subtidal and intertidal zones of tropical and temperate waters, are shaped by dynamic environmental conditions, including fluctuations in air and water temperature. Despite their polyphyletic origins, many seagrass species have independently evolved physiological and morphological adaptations that allow them to survive in highly variable conditions worldwide (den Hartog 1970). They persist in oscillating environments which can lead to fluctuations in the overall biomass and densities of seagrass, as well as affect their reproductive output (Wabnitz et al. 2008). However, both tropical and temperate seagrasses tend to exhibit optimal ranges for growth and photosynthesis (Niu et al. 2012). Even within these ranges, temperature fluctuations as well as extreme shifts pose a threat to seagrass acclimation and survival.

Of the many environmental factors influencing seagrass growth, productivity is mainly controlled by temperature, light and nutrient availability (Short 1987; Ralph et al. 2007; Collier et al. 2017). These key elements coupled with the hydrodynamics of a system affect the spatial distribution of seagrasses (Schanz and Asmus 2003; Uhrin and Turner 2018) and regulate biochemical processes that underpin growth and reproduction (Lee et al. 2007; Qin et al. 2020; Vercaemer et al. 2021). Several studies have been conducted to determine the effects of fluctuating temperatures (Campbell et al. 2006; Koch et al. 2007), light limitation (Koch and Erskine 2001; Minguito‐Frutos et al. 2023), extreme tidal exposure (Unsworth et al. 2012; Petrou et al. 2013), excess nutrients (Frankovich et al. 2009; Baggett et al. 2010), as well as their interactions with biodiversity (Blake and Duffy 2012; Eklöf et al. 2012) in a range of seagrass ecosystems. Such investigations, however, have been lacking in *Zostera capensis* habitats in South Africa. Despite a global surge in seagrass research, studies on *Z. capensis* and African systems remain notably scarce (Nguyen and Winters 2025). In addition to providing nursery habitat and stabilising sediments, *Z. capensis* plays a significant role in blue carbon dynamics. Recent assessments have quantified the species' carbon storage capacity in southern African estuaries, emphasising its potential contribution to climate mitigation efforts (Wasserman et al. 2023).

Anthropogenic activities have led to the intensification of eutrophic conditions in shallow coastal and estuarine environments, resulting in reduced availability of light and promoting anoxic (no oxygen) or hypoxic (low oxygen) conditions (Howarth et al. 2011). These conditions are amplified in warmer water where oxygen solubility is reduced (Shaffer et al. 2009), while respiration rates are enhanced (Quiñones‐Rivera et al. 2010; Rabalais et al. 2010). Prolonged anoxia leads to increased sulphide levels in the sediment, while hypoxia in the water column affects the internal oxygen conditions of seagrasses, both of which have detrimental effects on photosynthetic rates, metabolism and growth (Koch et al. 2007; Povidisa et al. 2009; Raun and Borum 2013). Die‐offs have been reported for seagrass meadows under hypoxic conditions (Holmer and Bondgaard 2001; Shields et al. 2018) and the suggested reasons for this include a combination of intrusion of sediment sulphide and anoxic plant tissue, exacerbated by a greater demand for oxygen in warmer water to meet respiratory needs (Mascaró et al. 2014; Koch and Erskine 2001; Koch et al. 2007). Since nearshore environments receive a higher input of organic matter from fluvial and estuarine sources than the open ocean, local‐scale variability in dissolved oxygen is higher in coastal compared with oceanic waters (Soetaert et al. 2006; Gilbert et al. 2010). Seagrasses are adapted anatomically and physiologically to contend with submersion as gases are efficiently diffused by well‐formed aerenchyma tissue in the leaves, and oxygen loss through the root system is well buffered (Papenbrock 2012; Brodersen et al. 2018). However, prolonged hypoxic and anoxic conditions retard growth (Holmer and Bondgaard 2001), resulting in the eventual loss of seagrass habitats (Hall et al. 2016). Restoration of these important systems depends on interventions that reduce eutrophication and improve water quality, which have been shown to increase the likelihood of seagrass recovery in some systems, although outcomes remain context‐dependent (van Katwijk et al. 2016; Orth et al. 2020).

In South Africa, there are a few remaining locations where the dominant seagrass species, the Cape dwarf‐eelgrass, *Zostera capensis* (*Nanozostera capensis* in the recent revision of Sullivan and Short 2023), forms monospecific stands creating essential habitat in estuaries and coastal environments. One notable example is Langebaan Lagoon, which supports multiple large stands of *Z. capensis* , listed by the IUCN (The World Conservation Union) as ‘Vulnerable’ because of its restricted range and declining population trend (Short et al. 2011). The extent of seagrass cover in this tidal lagoon has undergone changes over time. Estimated at ~58 ha in 1960 and reported to have declined to 22 ha by 2009, seagrass cover showed a marked recovery to 86 ha by 2014 (Adams 2016). To enhance our understanding of how *Z. capensis* , a temperate seagrass species, responds to fluctuating environments and anticipated climate extremes, it is important to comprehend its natural variability and identify the effects of key environmental factors. One way to achieve this is by assessing seasonal influences of morphometric parameters, which can shed light on the species' adaptive strategies and acclimation to different environmental conditions. Although numerous localised studies have emerged, a comprehensive global review on seagrass ecology has not been published in the past decade. This limits broad synthesis and highlights the value of region‐specific studies, particularly from underrepresented systems such as those in Africa.

This study firstly documents the spatial and temporal patterns of distribution in *Zostera capensis* in Langebaan Lagoon and identifies environmental factors influencing variability in seagrass density, biomass and morphometrics. This is a critical step in understanding natural and anthropogenic influences and is essential to establishing monitoring and restoration initiatives. Monitoring seagrass distributional patterns as well as tracking environmental parameters is a prerequisite to understanding natural dynamism while allowing for the detection of trends and anomalies. The prolonged effects of anomalies can cause shifts in ecosystem stable states, alter ecosystem function and lead to loss of productivity and biodiversity (Duarte 2002; Dennison 2009; Winters et al. 2011; Fraser et al. 2014). Knowledge of the influence of environmental factors therefore aids in identifying the effects of natural and human‐induced stress along with the effectiveness of conservation measures. Furthermore, results can be used to inform predictions of environmental effects under future climate change scenarios (Orth et al. 2006; Short et al. 2006). Here, morphologically distinct populations of *Z. capensis* were hypothesised to exhibit contrasting seasonal patterns in growth and structure, shaped by local environmental conditions and site‐specific exposure gradients within the lagoon. Specifically, cooler seasons were expected to support greater biomass and longer leaves, particularly in populations occupying deeper or less exposed intertidal zones, while warmer seasons were predicted to reduce seagrass density and productivity, especially in shallow, highly exposed sites.

Secondly, an experimental assessment of the thermal tolerance of *Zostera capensis* from two morphometrically distinct populations was carried out by subjecting them to four temperature treatments ranging from minimum to optimum to extreme (18°C, 22°C, 26°C and 30°C) under prolonged exposure. The central hypothesis is that continuous warming will drive *Z. capensis* beyond its physiological performance thresholds. The objective was to quantify changes in biomass, morphometrics, shoot density and algal fouling, thereby identifying temperature‐driven performance differences between morphotypes and predicting their potential for spatial expansion under future warming. Given the critical ecological role of seagrasses in primary production and habitat structuring, understanding morphotype‐specific resilience is key to forecasting their capacity to maintain ecological functions in shifting environments. Importantly, habitats dominated by distinct morphotypes support differing macrofaunal communities (Lawrence 2024), and only the long‐leaved morphotype shares genetic affinities with east coast populations (Phair et al. 2019), suggesting that morphotype expansion would have broader biodiversity implications. Three hypotheses were tested: (1) the small‐leaved morphotype, naturally exposed to temperatures > 28.5°C, would increase in growth metrics at 26°C–30°C, while the large‐leaved morphotype would perform best at cooler temperatures (22°C) and decline with warming; (2) epiphyte load would be higher under elevated temperatures (26°C and 30°C); and (3) large‐leaved morphotypes would exhibit greater algal fouling than their small‐leaved counterparts.

## 2 Methods

### 2.1 Study Area and Site Selection

**FIGURE 1:**
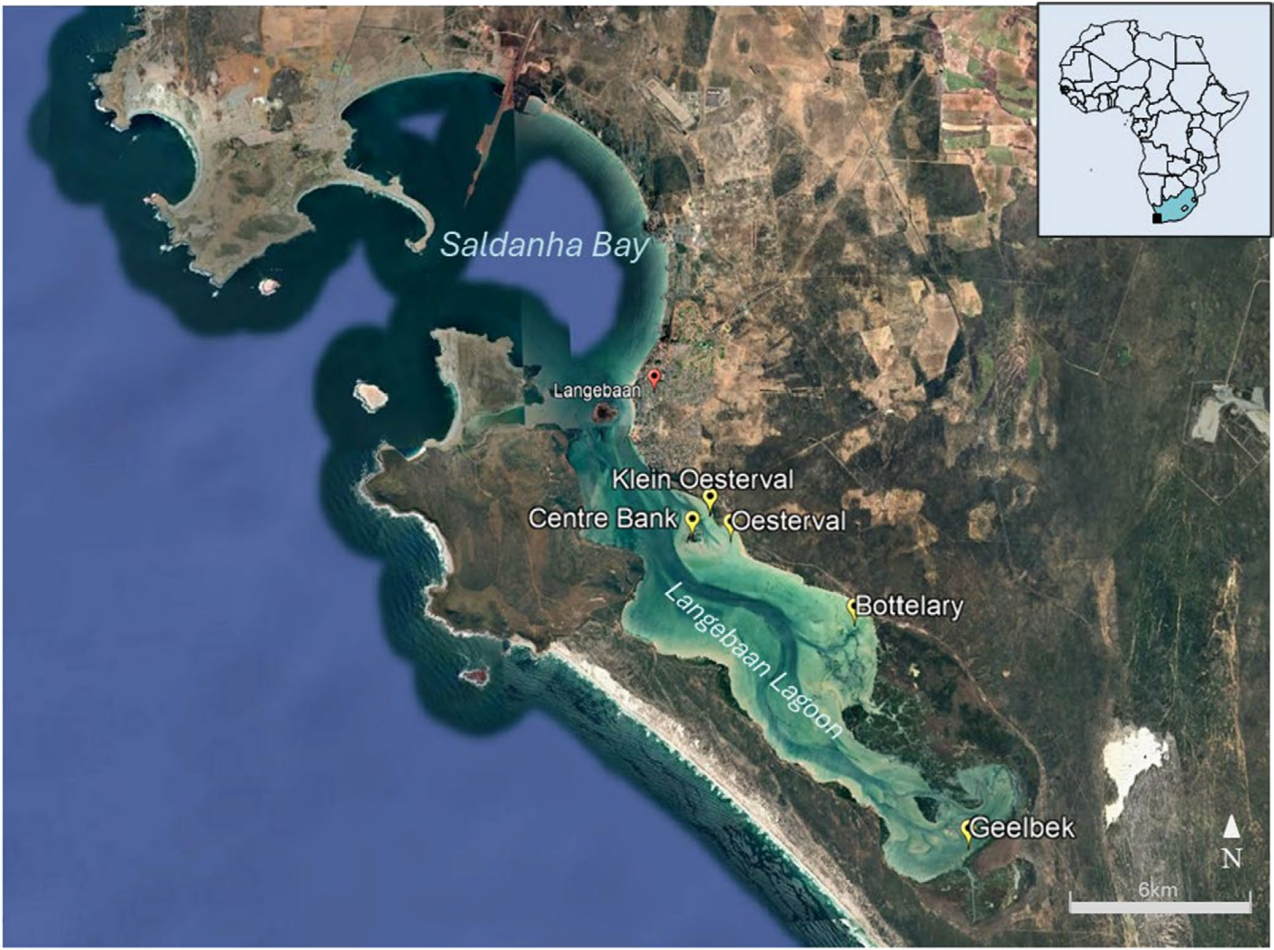
Langebaan Lagoon on the west coast of South Africa indicating the five major *Zostera capensis* sites sampled in this study (Google Earth Pro satellite image © 2020 Landsat/Copernicus).

Langebaan lagoon is a protected area located on the west coast of South Africa. At ~15 km in length and 4 km maximum width, it opens into the ocean through a large bay (Saldanha Bay) to the north that supports an industrial port (Figure 1). Benthic substrate in the lagoon comprises sandy sediment with macroalgae, salt marsh grass *Spartina* sp. and seagrass *Zostera capensis* as dominant macrophytes (Schils et al. 2001). The lagoon experiences semi‐diurnal tides and an average spring tidal range of 1.4 m (Day 1981). Seagrass populations were assessed at five sites (Centre Banks, Klein Oesterval, Oesterval, Bottelary and Geelbek; Figure 1) within the lagoon selected based on historical spatial descriptions (Day 1959) supported by satellite imagery (Landsat time‐lapse: 1984–2018) that confirmed these sites experienced consistent seasonal and/or interannual cover, albeit with a high degree of variability. Sites experienced different emergence periods during low tide, while the average water depth at high tide was estimated at 2 m at Centre Banks, 1.5 m at Klein Oesterval and Oesterval and 1 m at Bottelary and Geelbek (Flemming 1977). Maximal astronomical tidal range in South Africa is 2 m, as estuarine systems are microtidal (Whitfield 1992).

### 2.2 Seagrass Field Sampling

Since seagrass cover varied at each site, samples were taken according to the number of beds/patches present. This resulted in 10 beds being sampled at Centre Banks, Oesterval, and Geelbek, and five beds at Klein Oesterval and Bottelary for each season. To establish morphometric parameters of seagrasses, five cores (10 cm diameter × 15 cm; 0.008 m^2^) were randomly sampled in each bed during spring low tide when plants were completely exposed, over four austral seasons: autumn (April), winter (July), spring (October) and summer (January) (*N* = 520). Previous assessments describe the sediments of Langebaan Lagoon as largely homogeneous, dominated by fine to medium sands with low mud and organic content, conditions shaped by strong tidal flushing and mixing (Flemming 1977; Clark et al. 2012). Sediment characteristics across sites were therefore assumed to be comparable as no direct measurements were made in this study. Each core with intact shoots and roots/rhizomes was placed in a bag before being transported. In the laboratory, shoots and leaves were counted, and the average width and length of 80% of the leaves in each core were measured. Algal epiphytes were gently scraped off leaves into preweighed Petri dishes using the back of a scalpel blade. Shoots were separated from roots/rhizomes and dried at 60°C to a constant dry weight (~16 h) then weighed to obtain aboveground dry weight (g m^−2^). Epiphyte dry weight was measured after drying at 60°C until constant weight was reached.

### 2.3 Environmental Variables

Measurements of temperature, pH, turbidity, salinity, dissolved oxygen and chlorophyll *a* (chl *a*) were taken monthly at each site over 12 months during a similar time of day at spring high tide, using a handheld probe (YSI Multi Parameter Water Quality Sonde 6820 V2‐2V). During low tide, all sites were assumed to experience the same solar irradiance intensity; however, since seagrass beds occurred at varying positions along the shore, the exposure duration for which they were exposed varied. Turbidity was measured as a proxy for water column irradiance since suspended particulate matter can influence light levels reaching seagrass plants (Waycott et al. 2009; Collier et al. 2012). Sites were assigned an exposure level based on average emergence time during spring low tide coupled with distance (m) from the high water mark and classified as ‘high shore’ if it was within 10 m of the spring high water mark, ‘mid shore’ between 10 to 50 m, and ‘low shore’ if greater than 50 m. Exposure level was assigned as follows: Centre Banks—low shore, Oesterval—low/mid/high shore, Klein Oesterval—mid/low shore, Bottelary—high shore and Geelbek—high shore.

### 2.4 Experimental Temperature Selection

Results from field analyses informed an investigation into the effects of temperature on the growth of two morphotypes of *Zostera capensis*. Seagrass plants were subjected to four temperature treatments, that is, 18°C, 22°C, 26°C and 30°C in an indoor mesocosm experiment. A minimum temperature of 18°C was selected because sites that experienced the coolest temperatures (~18°C) were observed to produce the longest leaves. The maximum water temperature recorded in the lagoon during the study was 28.3°C, while average summer mean temperatures in both small‐leaved morphotypes (SLM) and large‐leaved (LLM) populations were 26.2°C and 21.8°C, respectively. Average maximum air temperature at Langebaan between 1973 and 2018 was 30.41°C ± 0.4 SE (SA Weather Service, unpub. data). The experiment, therefore, aimed to assess growth responses to optimal, average and maximal temperature ranges in this *Zostera* habitat. During the experiment, ambient temperatures ranged from 19.2°C to 21.9°C at LLM sites and 21.9°C to 24.2°C at SLM sites. Plants were, therefore, acclimatised at 20°C, which was considered acceptable for preventing thermal shock. Experiments were carried out during peak austral summer when water temperature ranges of 18°C to 24°C were recorded in 1 day at SLM sites, indicating that these plants can tolerate a temperature drop of up to 6°C. Furthermore, 18°C–20°C was used during 8‐ and 10‐week experimental trials, with growth responses observed in both morphotypes (Lawrence 2024).

### 2.5 Plant Preparation

Thirty‐six intact seagrass cores (10 cm diameter × 15 cm depth) comprising shoots, roots/rhizomes, and sediment were collected from Oesterval (LLM) and Geelbek (SLM) in seagrass beds of similar densities (*n* = 72). Each core was placed in a plant pot lined with a plastic bag and stored in a cooler during transportation to the aquarium. Upon arrival, all visible fauna were removed, and epiphytes were gently scraped off all leaves with the back of a scalpel blade. To prevent breakage and loss of leaves, shoots and roots/rhizomes were not separated. Instead, it was considered important to maintain intact above‐ and belowground biomass, which implied that shoot numbers varied between cores and averaged 20 ± 4.1 SE for LLM and 83 ± 3.2 SE for SLM.

### 2.6 Experimental Setup

Four mesocosms, one per temperature treatment, were set up. A mesocosm comprised three 80 L tanks pumped (AQUA H2O APH‐3000 pump) with filtered aerated seawater from a larger header 120 L tank, using an equal release flow system. Temperatures were adjusted to treatment levels using two 300 W aquarium heaters each in the 26°C and 30°C tanks and one each in the 18°C and 22°C tanks. Eight fluorescent tubes (Osram Lumilux Cool White 58 W/965) were mounted over each treatment (32 tubes in total) providing up to 400 μmol photons m^−2^ s^−1^ on a 12 h light/dark cycle. Despite being lower than natural sunlight, *Zostera* growth was sustained under these light conditions for up to 8 weeks during trial testing of the system (refer to Lawrence and Bolton 2023 for details on the experimental setup). Three replicate cores for each morphotype were placed in each tank (*n* = 9 cores × 2 morphotypes × 4 treatments). To account for potential tank effects, cores were randomly assigned to one of three replicate tanks per temperature treatment and repositioned weekly within tanks (see Figure A2: Appendix for a plot of the experimental design). Plants were held under saturating light conditions for 5 days then adjusted by 1°C per day until 18°C, 22°C, 26°C and 30°C were reached, and thereafter maintained for 4 weeks (Koch et al. 2007; Nejrup and Pedersen 2008; Chartrand et al. 2018). Treatment tanks were replenished weekly with fresh aerated seawater adjusted to treatment temperatures to maintain sufficient levels of nutrients and inorganic carbon to sustain seagrass growth (Nejrup and Pedersen 2008).

### 2.7 Morphological Measurements

Pretreatment measurements of shoot and leaf densities, leaf length, width, above and belowground biomass were taken for each morphotype from three cores at the start of the experiment. Pretreatment epiphyte biomass could not be obtained. On termination of the experiment, all cores were harvested, removed of sediment and the roots/rhizomes separated from shoots. Most of the leaves had detached from the shoots during this process, and the number of leaves per shoot could not be accurately estimated. Therefore, the total number of shoots and leaves per core (0.008m^2^) was counted. Seagrass morphometrics, including dry weight biomass of epiphytes and seagrass, were measured as described above.

### 2.8 Statistical Analyses

Strong collinearity emerged between temperature and pH (Spearman Rs = 0.87, *p* = 0.001), leaf and shoot densities (Spearman Rs = 0.92, *p* = 0.001) as well as leaf length and leaf width (Spearman Rs = 0.85, *p* = 0.001). Therefore, these variables were excluded from multivariate analyses so as not to confound the observed variation (Lipovetsky and Conklin 2001).

The effects of ‘season’ (fixed factor: four levels) and ‘site’ (fixed factor: five levels) on variation in four seagrass variables, that is, leaf length, shoot density, aboveground biomass (referred to as seagrass biomass) and epiphyte biomass were assessed using PERMANOVA+ for PRIMER (Version 6; Clarke and Gorley 2006). Seagrass variables were 4th root transformed to down weight the influence of large variances, and differences were calculated as Euclidean distances. In addition, two‐factor ANOVAs were performed to test the effect of ‘site’ and ‘season’ on each seagrass variable, followed by *post hoc* analyses (Tukey HSD).

To statistically relate the effects of environmental conditions to seagrass metrics, two‐tailed Pearson correlations were carried out between these variables over the seasonal average (3 months), compared to a 1‐month lag preceding the seagrass sampling event. One month was assumed to be a reasonable response time for a small‐leaved seagrass such as *Zostera capensis* since a similar species, *Z. noltei*, was observed to delay its response to seasonal fluctuations in this period relative to a large‐leaved species, *Z. marina* (Marbà et al. 1996). Correlations were higher between seagrass metrics and seasonal averages of environmental variables compared to measurements recorded 1 month prior (See Table A1: Appendix for correlation results), implying that seasonal environmental factors were better predictors of seagrass growth responses. This was the case for all environmental variables except dissolved oxygen, which showed a slightly stronger and significant correlation to leaf length and width in the preceding month (−0.58 and −0.61, respectively) than over the season (−0.47 and −0.53, respectively). Given that this difference was marginal, seasonal averages of all environmental variables were used to predict responses in seagrass metrics.

Environmental variables (water temperature, salinity, pH, turbidity, chlorophyll *a* and oxygen) were ‘normalised’ before analysis by subtracting the mean from each value for each variable and then dividing by their standard deviation. This brings environmental data that have different scales and units into proportion with each other to derive comparable outputs. Thereafter, two‐factor ANOVAs of each environmental variable across ‘sites’ and ‘seasons’ were performed to test for localised differences, followed by Tukey multiple comparisons *post hoc* analyses (Table A2).

Seagrass metrics were modelled using generalised additive mixed models (GAMMs) to identify potential predictors among five continuous environmental variables: water temperature, salinity, turbidity, dissolved oxygen and chlorophyll *a* and one categorical variable (shore height/exposure level). GAMMs are well‐suited for capturing nonlinear relationships due to their use of penalised regression splines, which incorporate smoothness selection in the estimation process (Wood and Augustin 2002; Wood 2017). Smoothness, expressed as ‘effective degrees of freedom’ (edf), reflects the complexity of the modelled curve, with higher edf values (> 8) indicating greater nonlinearity (Zuur et al. 2009; Wood 2017). Models were built with smoothers for continuous variables, using the mgcv package within the ‘nlme’ library in R 4.5. (Wood and Scheipl 2015; Pinheiro et al. 2016; R Development Core Team 2024). Knots were incrementally adjusted to produce biologically meaningful relationships and to avoid over‐specification (Wood 2017). Model assumptions were validated through diagnostic plots of residuals and random effects. The most parsimonious GAMMs were selected by assessing the significance of the random effect, minimising AIC values, maximising R^2^ values and retaining only significant predictor variables. Models used a Gaussian distribution with a maximum likelihood function (Zuur et al. 2009).

To test the effects of treatment temperatures, mixed‐effects ANOVAs were conducted in SPSS (IBM SPSS Statistics 28), with seagrass response variables as dependent variables, ‘treatment’ as a fixed factor, and ‘tank’ as a random factor nested within treatment. Each tank was assigned a single temperature level, and the nested structure allowed treatment effects to be isolated from variation associated with individual tanks. This was followed by post hoc (Tukey HSD) tests to assess differences between treatments. Seagrass metrics between small and large‐leaved morphotypes, as well as initial densities and biomass were highly varied; therefore, comparisons are presented in response variables pre‐ and post‐treatment within each morphotype and not between them. Thereafter, overall responses to temperature are discussed. Results are presented as values per core area (i.e., 0.008 m^2^).

Significance for all tests was assessed at *α* < 0.05, and variance is expressed as standard error (SE) of the mean. For each analysis, data were transformed (log or log *x* + 1) where necessary if assumptions for normality (Shapiro–Wilk's test) and homogeneity of variance (Levene's test) were not met.

## 3 Results

### 3.1 Spatial and Temporal Patterns in Seagrass Distribution

Contrasts in seagrass metrics were significant across sites, and the interaction of site and season contributed slightly more to the explained variation than site only (Table 1). Despite sites being > 90% similar in the multivariate space, a spatial gradient was visually evident with distance from the lagoon mouth (see Figure A1: Appendix for MDS results). Seasonal differences were not significant and accounted for the least variation (Table 1). A large percentage (51%) of multivariate variation was unexplained.

**TABLE 1:** PERMANOVA results based on Euclidean distances comparing seagrass variables (leaf length, shoot density, seagrass and epiphyte biomass) sampled across five seagrass sites over four seasons in Langebaan Lagoon.

Source of variation	df	MS	Pseudo‐F	*p*	Unique permutations	Effect size
Season	3	8.18	2.06	0.092	9943	17%
Site	4	10.46	20.66	**< 0.001**	9929	53%
Season × Site	12	3.03	5.99	**< 0.001**	9902	54%
Residual	79	0.51				51%

*Note:* Effect size was calculated from the estimated contribution of components of variation for each factor. *p* values are significant at *α* < 0.05 and in bold.

**FIGURE 2:**
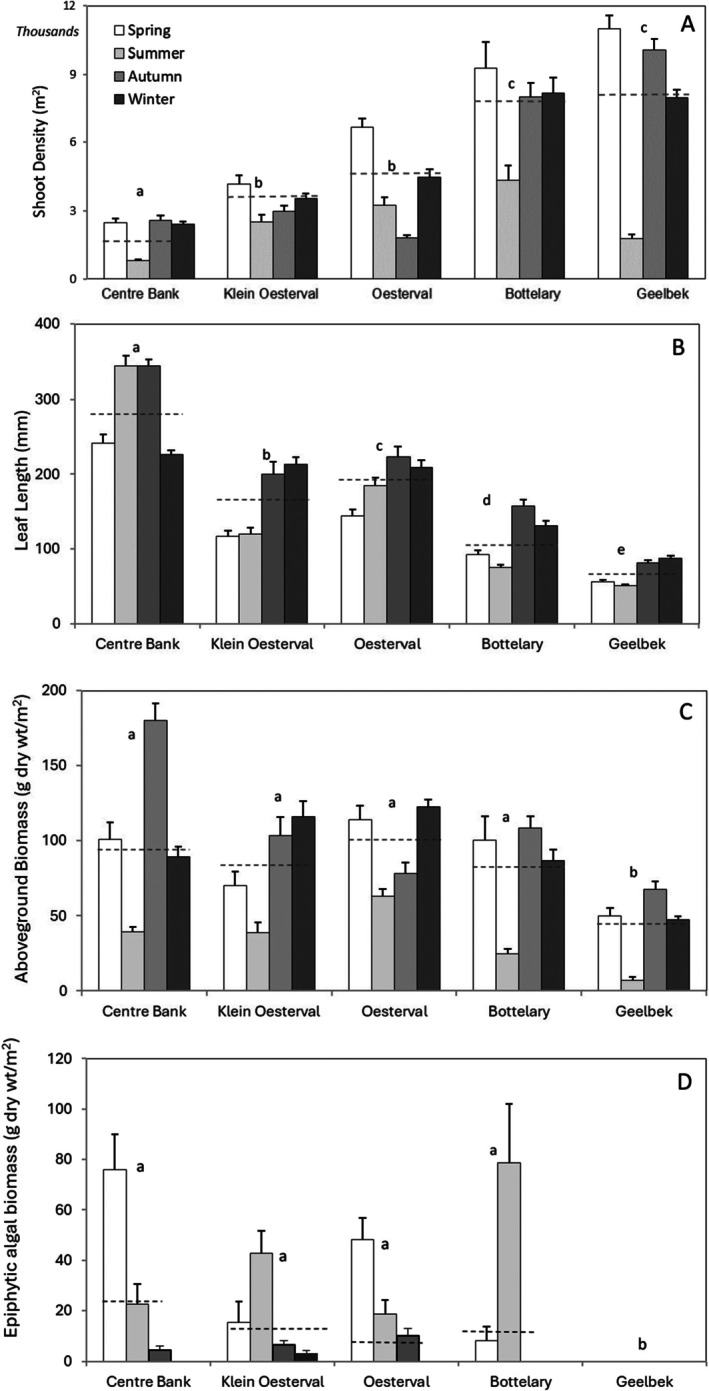
Mean shoot density (A), leaf length (B), aboveground biomass (C) and epiphyte biomass (D) of *Zostera capensis* habitats at five sites across four seasons in Langebaan Lagoon. No epiphytes were recorded at Geelbek. Small letters indicate similar means (dotted lines), not significantly different when compared using an a posteriori Tukey HSD test. Error bars represent +1 SE.

Shoot densities differed significantly across sites (*F*_4,494_ = 117.22, *p* < 0.001) and seasons (*F*_3,494_ = 75.5, *p* < 0.001). Bottelary and Geelbek had similar means, and the highest average densities of shoots compared to the other three sites (Figure 2A). Average shoot densities were also similar at Oesterval and Klein Oesterval, while seagrass beds at Centre Banks were the least dense (Figure 2A). The average lengths of seagrass leaves were also shorter at Geelbek and Bottelary compared to Centre Banks, Klein Oesterval and Oesterval (Figure 2B). Summer produced the lowest shoot densities across all sites except Oesterval which had lowest densities in autumn (Figure 2A). All sites generated longer leaves in autumn and winter, except Centre Banks where leaves were shortest in winter and longest in summer and autumn (Figure 2B). Average lengths of leaves differed significantly across sites (*F*_4,494_ = 179.49, *p* < 0.001) and seasons (*F*_3,494_ = 29.29, *p* < 0.001) due to variability in mean leaf lengths at all sites (Tukey HSD, *p* < 0.05).

Seagrass beds at Oesterval produced the highest average aboveground biomass (9.72 g dry wt m^−2^) across all seasons, followed by Centre Banks (9.45 g dry wt m^−2^) and Klein Oesterval (8.04 g dry wt m^−2^). Seagrass biomass at Bottelary was comparable to sites closer to the mouth, while biomass at Geelbek was consistently low over all seasons (Figure 2C). Seagrass biomass differed significantly between sites (*F*_4,494_ = 29.71, *p* < 0.001) and seasons (*F*_4,494_ = 57.23, *p* < 0.001) due mainly to high variability between Geelbek and the other four sites (Tukey HSD, *p* < 0.05). On average, seagrass biomass was lowest in summer in the lagoon and higher in autumn and winter (Figure 2C).

In general, epiphyte biomass did not correlate with leaf length or density. Epiphytes were found on seagrass leaves at Klein Oesterval over all seasons, while no algae were recorded at Oesterval, Centre Banks and Bottelary in winter, or from Geelbek during the entire sampling period (Figure 2D). On average, the highest epiphyte biomass was recorded on seagrass leaves at Centre Banks, followed by Bottelary, Oesterval and Klein Oesterval (Figure 2D). These differences were significant across sites (*F*_4,494_ = 7.12, *p* < 0.001), seasons (*F*_4,494_ = 22.03, *p* < 0.001) and their interaction (*F*_12,494_ = 8.22, *p* < 0.001) due to variation in mean epiphyte biomass between Geelbek and the other four sites (Tukey HSD, *p* < 0.05).

### 3.2 Spatial and Temporal Patterns in Environmental Variables

**FIGURE 3:**
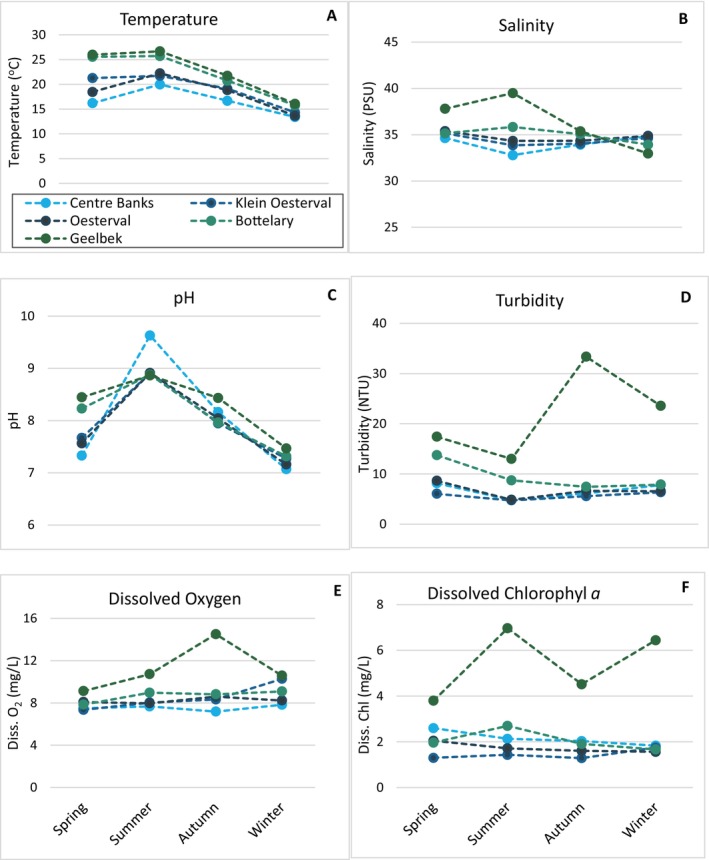
Average seasonal values of environmental variables recorded at five sites in Langebaan Lagoon.

Langebaan lagoon exhibits an environmental gradient from the mouth to the upper reaches of the lagoon. Average water temperature increased with distance from the mouth, with temperatures at Bottelary and Geelbek being consistently warmer compared to Klein Oesterval and Oesterval (Figure 3A). Water temperature at Centre Banks was the lowest on average throughout the study period, while the highest temperatures in the lagoon were recorded at Geelbek. Water temperature differences were significant across sites (*F*_4,141_ = 28.23, *p* < 0.001), seasons (*F*_4,141_ = 93.51, *p* < 0.001) and their interaction (*F*_12,141_ = 2.22, *p* = 0.015) explained mainly by variation in means between Centre Banks, Bottelary and Geelbek (Tukey HSD, *p* < 0.05, Figure 3A). Mean temperatures differed across all seasons except for spring and autumn (Tukey HSD, *p* < 0.05).

Salinity levels between sites (*F*_4,141_ = 13.39, *p* < 0.001), seasons (*F*_3,141_ = 8.74, *p* < 0.001) and their interaction (*F*_12,141_ = 9.75, *p* < 0.001) differed significantly, explained by the significantly higher salinity at Geelbek compared with the other four sites (Figure 3B, Tukey HSD, *p* < 0.05). Highest salinities were recorded at Geelbek and Bottelary, while Centre Banks, Klein Oesterval and Oesterval had lower and similar averages (Figure 3B). Seasonal variation was explained by the significant differences between salinities in summer and autumn, and summer and winter (Tukey HSD, *p* < 0.05).

Water column pH levels differed significantly between sites (*F*_4,141_ = 2.98, *p* = 0.022), seasons (*F*_3,141_ = 93.44, *p* < 0.001) and their interaction (*F*_12,141_ = 2.30, *p* = 0.011). This was mainly due to pH at Oesterval being significantly different from levels at Centre Banks, Geelbek and Bottelary (Figure 3C, Tukey HSD, *p* < 0.05). On average, pH at Geelbek was 0.33 levels higher, that is, more alkaline (8.2) compared with the other four sites; however, Centre Banks experienced the greatest range in pH, with a minimum of 6.9 in winter and a maximum of 9.7 in summer. All sites experienced alkaline conditions in summer compared to winter, except for Klein Oesterval, where alkalinity was higher in spring (Figure 3C).

Average water column turbidity was highest at Geelbek, with maximum levels reaching almost five times higher than those of other sites (Figure 3D). Average turbidity across the other four sites ranged from 5.73–8.57 NTUs. Turbidity differed significantly across sites (*F*_4,141_ = 22.37, *p* < 0.001) but not across seasons (*F*_3,141_ = 2.48, NS). The interaction between site and season was also significant (*F*_12,141_ = 2.55, *p* = 0.005) driven mainly by the high differences in turbidity between Geelbek and the other sites (Tukey HSD, *p* < 0.05, Figure 3D).

A generally high level of dissolved oxygen (DO) was recorded at Geelbek across all seasons, with the highest value of 26.44 mg/L recorded in autumn (Figure 3E). Centre Banks experienced the lowest DO levels, while DO at Klein Oesterval, Oesterval and Bottelary were similar (Figure 3E). DO between sites differed significantly (*F*_4,141_ = 6.75, *p* = 0.001) due to differences in means between sites closer to the mouth (Centre Banks, Oesterval and Klein Oesterval) and those closer to the head (Geelbek and Bottelary) of the lagoon (Tukey HSD, *p* < 0.05). Seasonal differences were not significant (*F*_3,141_ = 1.8, NS), while the interaction between sites and seasons was significant (*F*_12,141_ = 4.32, *p* = < 0.001).

Chlorophyll *a* (chl *a*) was significantly different at the site level (*F*_4,41_ = 20.76, *p* < 0.001) due to a higher mean recorded at Geelbek compared with the other sites (Tukey HSD, *p* < 0.05, Figure 3F). Average chl *a* was relatively stable at the other sites. No differences in chl *a* levels were observed between seasons (*F*_3,141_ = 1.089, NS) or the interaction between site and season (*F*_12,141_ = 1.013, NS).

**FIGURE 4:**
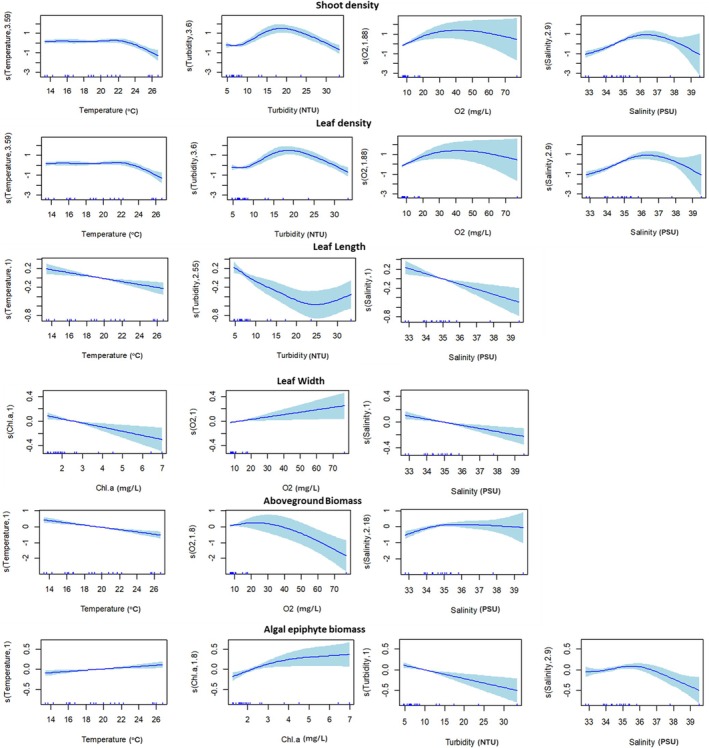
Plots of significant environmental predictors with fitted smooth terms showing linear and nonlinear relationships with six seagrass metrics from five sites in Langebaan Lagoon. Smoothed curves are represented by solid lines shaded by 95% confidence intervals. Short vertical lines (rug) on the *x*‐axis indicate actual observations for each variable forming the basis of the model's response. The *y*‐axis indicates the ‘component smooth’ centred on zero representing partial residuals from the model fit.

Results from generalised additive mixed modelling (GAMMs) showed patterns in seagrass densities in Langebaan Lagoon to be largely (> 80%) predicted by water temperature, turbidity, oxygen, salinity and exposure (Table 2). High seagrass densities were not supported in low and midshore positions; while densities were predicted to decline in water temperatures above 22°C (Figure 4).

**TABLE 2:** Summaries of generalised additive mixed models for top performing models predicting the responses of five seagrass metrics and epiphyte biomass to one categorical and five continuous environmental predictors.

	Parametric coefficients				Nonparametric smooth terms			
	Est	SE	*t*	*p*	Predictor	Edf	*F*	*p*
Shoot density (*R* ^2^ = 83%)								
Intercept	6.28	0.06	107.19	0.00	s(Temperature)	3.59	6.75	0.00
Exposure low	−0.66	0.10	−6.95	0.00	s(Turbidity)	3.60	13.41	0.00
Exposure mid	−0.64	0.10	−6.17	0.00	s(Oxygen)	1.88	8.41	0.00
					s(Salinity)	2.90	17.97	0.00
Leaf density (*R* ^2^ = 83%)								
Intercept	5.13	0.06	87.44	0.00	s(Temperature)	3.48	6.72	0.00
Exposure low	−0.64	0.09	−6.93	0.00	s(Turbidity)	3.62	12.98	0.00
Exposure mid	−0.62	0.08	−6.13	0.00	s(Oxygen)	1.64	8.36	0.00
					s(Salinity)	2.78	18.01	0.00
Leaf length (*R* ^2^ = 76%)								
Intercept	4.82	0.08	61.92	0.00	s(Temperature)	1.00	11.69	0.00
Exposure low	0.32	0.08	3.92	0.00	s(Turbidity)	2.55	5.85	0.00
Exposure mid	0.21	0.08	2.60	0.01	s(Salinity)	1.00	11.07	0.00
Leaf width (*R* ^2^ = 71%)								
Intercept	0.27	0.04	6.32	0.00	s(Chl *a*)	1.00	9.73	0.00
Exposure low	0.27	0.04	6.13	0.00	s(Oxygen)	1.00	5.62	0.02
Exposure mid	0.20	0.04	4.69	0.00	s(Salinity)	1.00	12.05	0.00
Aboveground biomass (*R* ^2^ = 69%)								
Intercept	−0.72	0.07	−9.72	0.00	s(Temperature)	1.00	22.78	0.00
					s(Oxygen)	1.80	6.92	0.00
					s(Salinity)	2.18	5.29	0.00
Epiphyte biomass (*R* ^2^ = 29%)								
Intercept	0.07	0.03	2.49	0.02	s(Temperature)	1.00	7.32	0.01
Exposure low	0.11	0.05	2.42	0.02	s(Chl *a*)	1.80	4.14	0.01
					s(Turbidity)	1.00	11.74	0.01
					s(Salinity)	2.90	3.55	0.02

*Note:* Estimates (Est) and standard errors (SE) of parametric coefficients are presented, along with approximate significance of thin plate regression spline smoother (s) terms and estimated degrees of freedom (Edf) for predictor variables. All variables are significant at *p* < 0.05.

Warmer water temperature and higher salinity are predicted to have a significant negative effect on leaf length; a response similarly projected for turbidity (Table 2). This is supported by observations of longer leaves at cooler temperatures and shorter leaves in more turbid conditions (Figure 2). Increasing salinity is also predicted to produce narrower leaves, while low and mid shore heights are predicted to support longer leaves (Figure 4).

Water temperature, oxygen and salinity were the principal factors influencing aboveground biomass, accounting for 69% of variation (Table 2). Exposure was not a significant predictor of aboveground biomass, whereas warmer temperature and higher oxygen are expected to negatively influence seagrass biomass (Figure 4).

Four environmental factors were significant in predicting patterns in epiphyte biomass (Table 2). Warmer temperature is predicted to favour epiphyte growth along with increasing levels of chl *a* (Figure 4). In contrast, decreasing water clarity and salinity are expected to negatively influence epiphyte growth. These variables explained only 29% of variation, implying that other factors, possibly biotic and abiotic, are likely better predictors of epiphyte growth.

### 3.3 Experimental Responses to Temperature

#### 3.3.1 Large‐Leaved Morphotype

**FIGURE 5:**
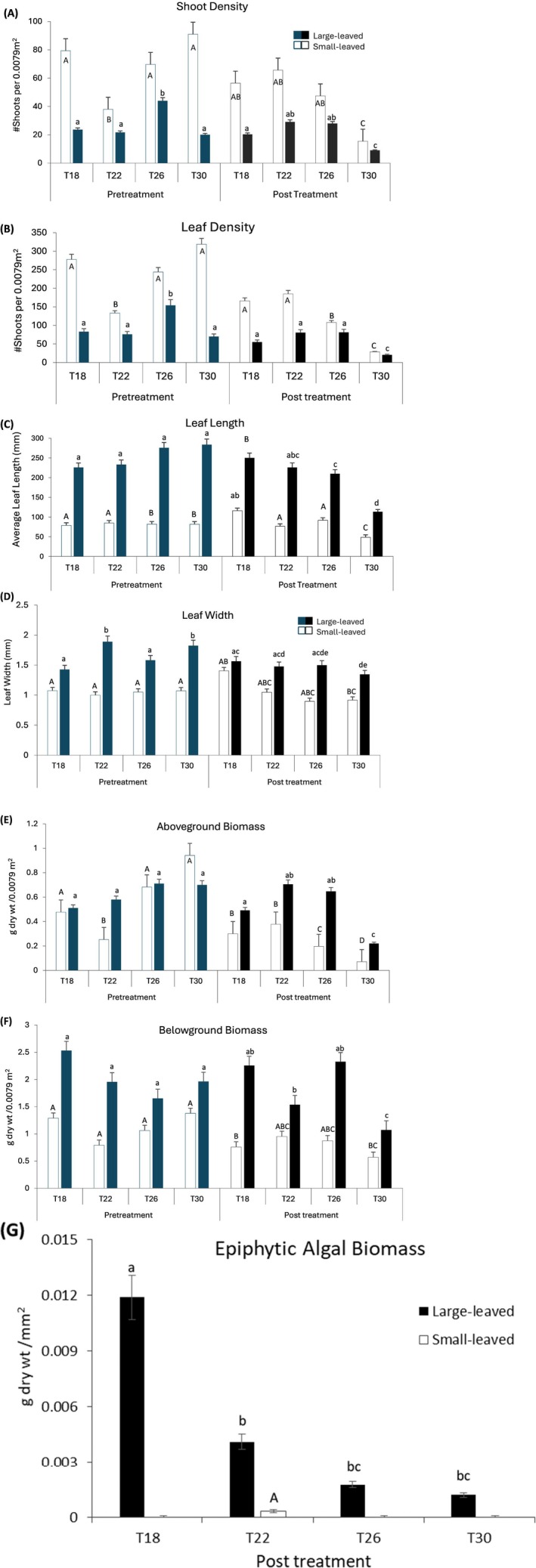
Variation in shoot (A) and leaf densities (B), leaf length (C), leaf width (D), aboveground (E), belowground (F) and epiphytic algal biomass (G) of small‐leaved (white fill) and large‐leaved (solid fill) morphotypes of *Zostera capensis* pre (blue bars) and post‐treatment (black bars) in T18°C, T22°C, T26°C and T30^o^C temperatures. Bars show means ±SE. Large and small letters represent homogenous means not significantly different (*p* > 0.05) for small‐ and large‐leaved morphotypes respectively, when compared in a Tukey HSD test.

Average shoot densities for large‐leaved morphotypes (LLM) were initially highest in the 26°C treatment (Figure 5A), with no significant differences observed across treatments after the experiment (Table 3). Pretreatment densities remained stable at 22°C and 26°C but were significantly different from those at 18°C and 30°C. Leaf densities, strongly correlated with shoot densities (Spearman Rs = 0.89, *p* < 0.001), showed significant variation across treatments (*F*_4,10_ = 4.65, *p* = 0.02). Significant tank effects were also detected for some traits; however, treatment temperature explained a substantial proportion of the variance (*p* < 0.05; Table 3). Due to challenges in estimating leaves per shoot and variations in shoot and leaf densities, changes in leaf densities were attributed primarily to differences in the number of leaves per shoot. Post hoc analyses revealed significant differences in leaf densities among treatments, except between 22°C and 26°C (Figure 5B).

**TABLE 3:** ANOVA results on the effect of treatment (fixed factor: four temperature levels) and tank (random factor nested within treatment) on shoot and leaf densities, leaf length and width, above (AG) and below ground (BG) biomass and epiphyte biomass of two morphotypes (large‐ and small‐leaved) of *Zostera capensis* seagrass.

Variable	Factor	Large‐leaved			Small‐leaved		
		F_4,10_	*p*	Partial Eta^2^	F_4,10_	*p*	Partial Eta^2^
Shoot density	Treatment	2.10	0.16	46%	0.73	0.59	23%
	Tank (Treatment)	158.76	**0.00**	78%	47.29	**0.00**	53%
Leaf density	Treatment	4.65	**0.02**	65%	7.93	**0.00**	76%
	Tank (Treatment)	135.88	**0.00**	76%	27.62	**0.00**	39%
Leaf length	Treatment	13.24	**0.00**	84%	11.68	**0.00**	82%
	Tank (Treatment)	3.84	**0.00**	8%	12.29	**0.00**	22%
Leaf width	Treatment	2.29	0.13	48%	3.43	**0.05**	58%
	Tank (Treatment)	2.24	**0.02**	5%	26.79	**0.00**	39%
AG biomass	Treatment	1.06	0.42	30%	1.19	0.37	32%
	Tank (Treatment)	34.64	**0.00**	44%	30.88	**0.00**	42%
BG biomass	Treatment	5.03	**0.02**	67%	4.69	**0.02**	65%
	Tank (Treatment)	5.35	**0.00**	11%	4.16	**0.00**	9%
Epiphyte biomass	Treatment	1.22	0.36	31%	2.98	0.10	53%
	Tank (Treatment)	13.07	**0.00**	23%	8.45	**0.00**	17%

*Note:* Effect size of each variable on the observed variation is represented by partial Eta^2^ values. Significant effects are based on *p* =/< 0.05 and are emboldened.

Leaf lengths increased at 18°C compared to pretreatment values, remained unchanged at 22°C, and declined in warmer treatments (26°C and 30°C), with significant treatment effects (*F*_4,10_ = 13.24, *p* = 0.001) (Figure 5C). Differences in leaf lengths between pretreatment, 18°C, and 22°C were not significant, but 26°C showed significant declines compared to pretreatment values. Significant differences were also observed between 18°C and 30°C. Post hoc analyses indicated that leaf lengths at 22°C and 26°C were similar, with the shortest leaves recorded at 30°C (Figure 5C). In contrast, leaf widths remained largely consistent across all treatments except 30°C, where significant variation was noted compared to pretreatment and 18°C values. Treatment effects on leaf width were not statistically significant (Table 3), with widths at 22°C and 26°C remaining comparable (Figure 5D). This indicates that temperature had a more pronounced effect on leaf length than width, particularly at higher temperatures.

Exposure to supraoptimal temperatures (30°C) caused a substantial decline in aboveground biomass (AGB), though treatment effects were not statistically significant (Table 3). Post hoc analysis revealed significant differences in AGB between 18°C and the other three treatments. AGB levels at 22°C and 26°C were similar and significantly higher than that at 30°C (Figure 5E). Belowground biomass (BGB), however, showed significant treatment effects (*F*_4,10_ = 5.03, *p* = 0.02) and remained consistent pre‐ and post‐treatment at 18°C and 26°C (Table 3). Post hoc tests indicated significant declines in BGB at 30°C compared to all other treatments, highlighting a marked reduction following exposure to warming (Figure 5F).

Epiphyte biomass per leaf area was highest in the 18°C treatment and showed a declining trend with increasing temperature (Figure 5G). While significant differences in epiphyte biomass were observed between 18°C and all other treatments, the overall effect of temperature on epiphyte biomass variation was not statistically significant (Table 3).

#### 3.3.2 Small‐Leaved Morphotype

Shoot densities of small‐leaved morphotypes (SLM) showed a similar response to temperature as that of LLM. Although average shoot densities were generally higher pre‐ compared with post‐treatment (Figure 5A), differences between treatments were not statistically significant (Table 3). Pretreatment densities at 18°C, 22°C and 26°C remained consistent post‐treatment, whereas shoot densities at 30°C showed significant differences. In contrast, temperature effects on SLM leaf densities were significant (*F*_4,10_ = 7.93, *p* < 0.01; Table 3). Significant tank effects (*p* < 0.05) were also observed for some traits, but treatment temperature remained a key driver of trait variation. As with LLM, variations in leaf densities were primarily driven by changes in the number of leaves per shoot, although precise estimates were unavailable. Leaf densities declined post‐treatment across all temperatures, with significant differences observed between treatments, except between 22°C and 26°C (Figure 5B).

In SLM treatments, leaf lengths increased at 18°C and 26°C compared with pretreatment levels, remained stable at 22°C, and decreased at 30°C (Figure 5C). Significant treatment effects on leaf lengths were detected (*F*_4,10_ = 11.68, *p* = 0.001; Table 3). These effects were primarily driven by differences between pre‐ and post‐treatment lengths at 26°C and 30°C, as well as between 18°C and the warmer treatments. Leaf widths were generally consistent pre‐ and post‐treatment across most temperatures, except 30°C, where a notable decline was observed, and 18°C, which showed an increase compared with pretreatment levels (Figure 5D). Significant treatment effects on leaf width (*F*_4,10_ = 3.23, *p* = 0.05) were attributed to differences observed at 30°C compared with other treatments.

Aboveground biomass (AGB) of SLM treatments increased at 22°C compared with pretreatment levels, remained stable at 18°C and 26°C, and declined significantly at 30°C (Figure 5E). While the overall treatment effect was not statistically significant (Table 3), post hoc analysis revealed significant variation in AGB between 18°C and the other treatments, with no significant pre‐ and post‐treatment differences except at 30°C. AGB showed similar patterns of variation at 22°C and 26°C. Belowground biomass (BGB) increased at 26°C compared with pretreatment levels but remained similar across most treatments, except 30°C, which produced the lowest BGB (Figure 5F). Post hoc tests identified significant differences in BGB between pre‐ and post‐treatment at 22°C and 30°C. Additionally, significant variation was observed between 18°C and 22°C, 22°C and 26°C, and across all treatments compared with 30°C.

Leaf scrapings from SLM leaves revealed no detectable epiphytes in the 18°C, 26°C, and 30°C treatments, with only a minimal amount (0.0003 g dry weight mm^−2^) collected at 22°C (Figure 5G). Since pretreatment epiphyte biomass measurements were unavailable, direct changes pre‐ and post‐treatment could not be evaluated; however, field observations indicated a general absence of epiphytes on SLM leaves (Figure 2D). Post hoc analyses revealed that epiphyte biomass was similar among the 22°C, 26°C and 30°C treatments but significantly different from 18°C.

## 4 Discussion

In Langebaan Lagoon, seagrass growth showed marked variation across sites and seasons, largely due to differences between stands closer to the mouth (Centre Banks, Klein Oesterval, and Oesterval) and those further up the lagoon (Bottlery and Geelbek). Environmental conditions varied significantly between these sites, but not consistently across seasons, indicating that local‐scale environmental conditions may exert a stronger influence on seagrass dynamics than broader seasonal trends. These findings suggest that fine‐scale drivers, such as tidal exposure, turbidity and water retention, may override seasonal or climatic patterns. This aligns with other studies showing that local hydrodynamics and light attenuation can have a greater impact on growth responses than temperature or photoperiod alone (Ricart et al. 2020; Hensel et al. 2024). Such results support the potential effectiveness of localised conservation efforts to buffer against warming, eutrophication and sedimentation.

### 4.1 Temporal Patterns and Environmental Stress

The densest stands of *Zostera capensis* were recorded in early summer, declining significantly by late summer. These declines coincided with supraoptimal temperatures and light levels, consistent with patterns observed in other temperate seagrass species that peak in spring and early summer before senescing due to heat and light stress (Qiu et al. 2017; Delgado‐Serrano and Tuya 2023). Biomass and shoot density markedly declined particularly at Geelbek and Centre Banks during late summer, followed by signs of recovery in late autumn as temperatures and irradiance decreased. This rebound suggests that *Z. capensis* may employ a seasonal dieback strategy, relying on energy reserves in belowground tissues to regenerate once conditions improve (Adams and Bate 1994). Similar poststress recovery patterns have been observed in *Z. marina* following heat exposure (Deguette et al. 2022), reinforcing the role of thermal plasticity in maintaining resilience (Duarte et al. 2006; Lee et al. 2007; McDonald et al. 2016).

At Centre Banks, submergence during spring and neap tides and year‐round cooler temperatures (13°C–20°C) supported higher biomass and longer leaves. The site's position as the first to be submerged by the incoming tide results in minimal exposure during low tides (Day 1959, pers. observation). These conditions approximate laboratory‐derived optima for *Z. capensis* (15°C–20°C; Edgecumbe 1980) and align with thermal preferences for other *Zostera* species (Nejrup and Pedersen 2008; Beca‐Carretero et al. 2018). In contrast, Geelbek experienced higher peak temperatures (> 28°C), longer emergence periods and elevated turbidity, likely contributing to greater stress and reduced productivity. Elevated temperatures increase respiration and inhibit photosynthesis, shifting the metabolic balance towards decline (Kim et al. 2012; Rasmusson et al. 2020).

### 4.2 Environmental Controls and Spatial Patterns

Spatial, rather than temporal, variation was more pronounced in environmental parameters such as turbidity, exposure, pH and temperature. Among these, turbidity and exposure emerged as the strongest predictors of seagrass trait variation, jointly accounting for 66% of spatial differences. Geelbek, characterised by high turbidity, elevated salinity and temperature and shallow water, consistently supported a small‐leaved morphotype adapted to upper intertidal stressors such as high desiccation and low irradiance (Björk et al. 1999; Zabarte‐Maeztu et al. 2021). Conversely, sites closer to the mouth with more stable submersion and water flow supported large‐leaved morphotypes with greater biomass and leaf length. These differences are likely due to phenotypic plasticity rather than genetic differentiation, given the high connectivity among populations (Phair et al. 2019; Lawrence and Bolton 2023).

All sites exhibited lower pH in winter, a common estuarine trend associated with increased rainfall and nutrient input in Mediterranean climates (Daniel et al. 2013; Fietzke et al. 2015). Reduced pH (i.e., elevated CO_2_ availability) can enhance seagrass photosynthesis, especially when HCO_3_^−^ (bicarbonate) uptake becomes energetically costly under low‐light conditions (Koch et al. 2013; Cox et al. 2015; Borum et al. 2016). The negative correlation between biomass and pH suggests that mild acidification may benefit *Z. capensis* in Langebaan. However, seagrasses and other autotrophs can modify local pH through metabolic processes and calcification (Hofmann et al. 2011; Duarte et al. 2013; Mucci 1983; Hurd et al. 2009), resulting in high spatial and temporal variability, making long‐term high‐resolution monitoring critical to interpreting these complex interactions (Saderne et al. 2013).

Like pH, dissolved oxygen (DO) varied at fine spatial scales (Felisberto et al. 2015). Although seagrass size and biomass were lower at Geelbek, DO levels were generally higher than at mouth‐proximal sites. While exposure and turbidity were the primary drivers of seagrass variation, DO emerged as the third most important predictor of density and biomass in the GAMM analysis. This likely reflects the influence of both photosynthetic activity and land‐based runoff enriched with organic matter from adjacent salt marshes (Silva et al. 2009; Whitfield 2005).

Geelbek consistently had the highest values for temperature, salinity, turbidity, DO, and chlorophyll *a*, combined with prolonged emergence and shallow depth. These conditions favoured a narrow, fringing zone of small‐leaved seagrass likely constrained by light availability and desiccation tolerance. Suspended sediments were a primary stressor at this site, influencing light penetration, sediment dynamics and rhizosphere chemistry (Zabarte‐Maeztu et al. 2021). The persistence of seagrasses in this zone, despite thermal and desiccation stress, underscores a greater tolerance to aerial than subaqueous low‐light conditions (Björk et al. 1999; Kim et al. 2020).

### 4.3 Plasticity and Morphological Adaptation

Phenotypic plasticity was most apparent in shoot size and leaf traits, with small‐leaved morphotypes (SLMs) dominating shallow, turbid and highly exposed areas. These smaller forms likely reduce desiccation risk through compact architecture, faster turnover and closer contact with moist sediment during low tide (Tanaka and Nakaoka 2004; Shafer et al. 2007; Björk et al. 1999). Comparable patterns have been reported for *Z. marina* , *Halophila ovalis* , *Halodule wrightii* and others across intertidal–subtidal gradients (Peralta et al. 2000; Cabaço et al. 2009; Kaewsrikhaw et al. 2016).

Large‐leaved morphotypes (LLMs) confined to deeper low‐shore zones with higher flow velocities and clearer waters likely benefit from improved gas exchange and nutrient uptake (Cornelisen and Thomas 2006; Weitzman et al. 2013; Peralta et al. 2021). Conversely, SLMs are better suited to upper shorelines where burial, high temperature and desiccation stress are common (Zabarte‐Maeztu et al. 2021; Kim et al. 2020). However, recent work suggests plasticity alone may not be sufficient under multistressor conditions involving heat, light and desiccation (Entrambasaguas et al. 2021; Bertelli et al. 2021). The reduced size and compact form of SLMs reflect trade‐offs between minimising desiccation and UV exposure and photosynthetic efficiency. Genetic data indicate high connectivity and low divergence among morphotypes, supporting the view that observed morphological differences are environmentally induced (Phair et al. 2019). This is consistent with other systems, including *Z. marina* in the Wadden Sea and *Posidonia oceanica* in the Mediterranean, where intraspecific variability arises from gene regulation and epigenetic processes (Oetjen and Reusch 2007; Procaccini et al. 2017).

### 4.4 Experimental Insights Into Thermal Thresholds and Trait Shifts

Under experimental conditions, both morphotypes performed best at 22°C. Above 26°C, shoot density, leaf length and biomass declined, with neither morphotype performing well at 30°C. Surprisingly, SLMs did not outperform LLMs at 30°C, despite their natural exposure to higher temperatures in situ suggesting both types are near thermal tolerance limits. LLMs increased belowground biomass at 26°C, potentially as a compensatory mechanism under stress, although this may limit productivity long term (Marín‐Guirao et al. 2018). Rapid shoot loss at 30°C suggests that heat stress overcame any adaptive advantages, reinforcing findings from other *Zostera* systems, where productivity and survival drop sharply above 22°C–26°C (Nejrup and Pedersen 2008; Edgecumbe 1980; Höffle et al. 2011). When desiccation stress was removed, SLMs increased leaf length and density, indicating capacity for plastic adjustment. This aligns with findings by Apichanangkool and Prathep (2014), where intertidal seagrasses adjusted morphology in response to stress relief. However, this plasticity is not unlimited; continued warming may drive populations beyond physiological thresholds (Pedersen et al. 2016; Rasmusson et al. 2020). Recent metabolomic analyses show that species‐specific biochemical responses to combined heat and low‐light stress, such as those during marine heatwaves, can compromise recovery and lead to rapid biomass loss (Jung et al. 2023), reinforcing the vulnerability of even thermally tolerant forms like *Zostera* under compound stress.

Significant tank or block effects were observed for several response variables that are not uncommon in mesocosm experiments, especially with sensitive species such as seagrasses (Spivak et al. 2011; Hammerschlag‐Peyer et al. 2013; Tew et al. 2021). A schematic of the experimental layout (Figure A2) illustrates the spatial arrangement and design controls employed in the experiment. Despite tank‐level variation, treatment temperature accounted for a substantial proportion of variance in multiple traits, particularly leaf length, leaf density, and belowground biomass, confirming the validity of these results.

Contrary to expectations, epiphyte load declined at higher temperatures, particularly in SLMs. While high temperatures can stimulate algal growth, this response was absent, suggesting additional constraints. Possible explanations include host‐specific traits such as altered nutrient competition or chemical defences such as phenolic compounds or condensed tannins that inhibit algal settlement (Pohnert 2004; Lane and Kubanek 2008; Ross et al. 2008). The absence of grazers and initial cleaning of seagrass leaves suggests these differences were not driven by external biotic factors. While reduced epiphytes may lower shading stress, they also limit food availability for grazers, potentially altering trophic interactions (van Montfrans et al. 1984; Jaschinski and Sommer 2011). Field observations at Geelbek suggest strong top‐down grazer control also limits algal growth (Lawrence and Bolton 2023).

### 4.5 Implications for Resilience and Management

Together, field and experimental results suggest that *Z. capensis* exhibits notable plasticity but limited capacity to tolerate sustained warming. LLMs, while more productive, may be displaced or outcompeted by SLMs under warming scenarios; however, SLMs also approach thermal limits near 30°C. Such shifts may reduce functional diversity, canopy complexity, and ecological services, for example, reduced sediment stabilisation, carbon sequestration and faunal support (Wasserman et al. 2023). Intrinsic factors such as energy allocation, turnover rates and photosynthetic plasticity will likely influence population outcomes; however, even high plasticity may not suffice if warming trends continue unchecked (Procaccini et al. 2017; Bertelli et al. 2021; Entrambasaguas et al. 2021).

Management should therefore prioritise reducing local stressors like turbidity, eutrophication and sedimentation, to mitigate warming impacts. Conserving Langebaan's seagrass habitats contributes not only to local biodiversity and ecological function but also to global climate and conservation goals through carbon storage and habitat provisioning (Duarte et al. 2025). Hydrodynamic factors such as flow velocity, water retention and wave exposure should also inform management as they influence nutrient availability and gas exchange (Delgard et al. 2016; Gillis et al. 2017). These conditions interact with temperature to shape morphological traits. Higher flushing rates and stable submersion near the lagoon mouth may support taller seagrass stands, creating potential refugia for LLMs if adequately protected (Stevens and Lacy 2012; Pace et al. 2017).

## 5 Conclusions

Marked morphological and physiological differences in *Zostera capensis* across Langebaan Lagoon reflect plastic responses to localised environmental conditions. Both morphotypes perform optimally at 22°C, with declines evident above 26°C, and physiological thresholds exceeded at 30°C. Warming is likely to favour SLMs temporarily, but their tolerance is also limited, risking overall ecosystem simplification and functional loss. Conservation must integrate field observations with experimental insights to refine predictive models and inform climate‐smart management. Future strategies should identify and protect thermal refugia, reduce compounding stressors, and consider adaptive interventions such as genetic sourcing and targeted restoration. As climate extremes intensify, adaptive, forward‐looking conservation is essential to preserve *Z. capensis* and the ecosystems it supports (Daru and Rock 2023; Entrambasaguas et al. 2021).

## Author Contributions

**Cloverley M. Lawrence:** conceptualization (lead), data curation (lead), formal analysis (lead), investigation (lead), methodology (lead), project administration (lead).

## Conflicts of Interest

The author declares no conflicts of interest.

## Supporting information


**Appendix S1:** ece372011‐sup‐0001‐Appendix.docx.

## Data Availability

All data supporting the findings of this study are openly available at https://doi.org/10.5281/zenodo.15647740. Additional supporting figures and tables are provided in the Appendix.
