# Supplementary material for: Trait Plasticity and Warming Vulnerability in a Structurally Diverse Seagrass Ecosystem
Source: Ecol Evol. 2025 Aug 21;15(8):e72011. doi: 10.1002/ece3.72011 (PMC12370319; doi:10.1002/ece3.72011)
Supplement: Supplementary file 1 — Appendix S1: ece372011‐sup‐0001‐Appendix.docx. [file ECE3-15-e72011-s001.docx]

**Appendix**

**Table A1:** Pearson 2-tailed correlations between seagrass morphological metrics and environmental variables differentiating responses based on a 3 month seasonal average and 1 month lag preceding seagrass sampling. Correlations significant at α < 0.05 are in bold.

|  | 3 Month seasonal average | | | | | |  | 1 Month lag | | | | | |
| --- | --- | --- | --- | --- | --- | --- | --- | --- | --- | --- | --- | --- | --- |
|  | Temp | Sal* | Turb** | pH | O_2_ | Chl *a* |  | Temp | Sal* | Turb** | pH | O_2_ | Chl *a* |
| Seagrass biomass | **-0.53** | **-0.25** | **-0.23** | **-0.53** | **-0.28** | **-0.46** |  | **-0.45** | -0.09 | **-0.23** | **-0.40** | **-0.37** | **-0.45** |
| Shoot density | 0.15 | 0.21 | **0.67** | -0.24 | **0.49** | **0.31** |  | 0.08 | **0.30** | **0.59** | **-0.34** | **0.31** | 0.19 |
| Blade density | 0.18 | 0.19 | **0.61** | -0.17 | **0.41** | **0.25** |  | 0.06 | **0.25** | **0.52** | **-0.26** | **0.39** | 0.11 |
| Leaf length | **-0.51** | **-0.55** | **-0.53** | -0.02 | **-0.47** | **-0.50** |  | **-0.37** | **-0.46** | **-0.44** | 0.08 | **-0.58** | **-0.42** |
| Leaf width | **-0.41** | **-0.47** | **-0.56** | -0.09 | **-0.53** | **-0.56** |  | **-0.37** | **-0.39** | **-0.51** | 0.02 | **-0.61** | **-0.50** |
| Epi-algal biomass | 0.05 | -0.05 | -0.18 | 0.04 | **-0.24** | -0.14 |  | -0.01 | 0.08 | -0.20 | -0.04 | -0.14 | -0.19 |

*Sal = Salinity

**Turb = Turbidity

***Lagoon Head Lagoon Mouth***a

**A**

**B**

**Fig. A1:** Multi-dimensional scaling ordination of distances among centroids based on Euclidean distances for site (A) nested within season (B) distributions of morphometrics and associated epiphyte biomass of Zostera capensis in Langebaan Lagoon.

**Table A2:** Abiotic variables recorded at five sites in Langebaan Lagoon. Means ± 1 standard error, minimum and maximum values are presented. Small letters represent homogenous means between sites that are not significantly different when compared using Tukey HSD testing.

|  | **Centre Banks** | **Klein Oesterval** | **Oesterval** | **Bottelary** | **Geelbek** |
| --- | --- | --- | --- | --- | --- |
| **Water temperature (^o^C)** | 15.86^a^ ±0.46 | 18.64^abc^ ±0.75 | 17.89^ab^ ±0.65 | 20.74^bc^ ±0.93 | 21.42^c^ ±0.92 |
|  | 12.68 | 13.62 | 13.07 | 14.68 | 15.01 |
|  | 20.15 | 25.53 | 23.78 | 27.68 | 28.49 |
| **Salinity** | 34.26^a^ ±0.21 | 34.40 ^a^ ±0.21 | 34.71^a^ ±0.20 | 34.86 ^a^ ±0.29 | 35.76 ^b^ ±0.55 |
|  | 32.38 | 32.40 | 32.80 | 32.30 | 31.87 |
|  | 35.80 | 36.30 | 36.13 | 37.32 | 40.65 |
| **Ph** | 7.78^a^ ±0.19 | 7.88^bc^ ±0.13 | 7.86^c^ ±0.14 | 7.98^ab^ ±0.13 | 8.21^a^ ±0.13 |
|  | 6.88 | 7.09 | 7.06 | 7.08 | 7.31 |
|  | 9.67 | 9.48 | 9.48 | 9.32 | 9.28 |
| **Turbidity**  **(nephelometric turbidity units)** | 6.99^a^ ±0.58 | 5.73^a^ ±0.39 | 6.66^a^ ±0.45 | 8.57^a^ ±0.69 | 23.79^b^ ±3.07 |
|  | 4.40 | 3.60 | 4.30 | 5.10 | 7.70 |
|  | 16.10 | 12.90 | 14.00 | 18.00 | 73.30 |
| **Oxygen (mg/litre)** | 7.52^a^ ±0.16 | 8.66^ab^ ±0.26 | 8.26^ab^ ±0.70 | 8.83^b^ ±0.27 | 11.76^c^ ±1.92 |
|  | 6.17 | 6.84 | 6.24 | 7.32 | 7.32 |
|  | 8.40 | 16.40 | 9.60 | 10.65 | 26.44 |
| **Chlorophyll *a* (mg/litre)** | 2.10^a^ ±0.21 | 1.46^a^ ±0.11 | 1.71^a^ ±0.10 | 2.01^a^ ±0.16 | 5.63^b^ ±0.69 |
|  | 1.00 | 0.70 | 1.00 | 0.90 | 1.70 |
|  | 7.30 | 3.40 | 3.60 | 4.10 | 15.50 |
| **Exposure/shore height (m)** | Low shore | Low/mid/high shore | Mid/low shore | High shore | High shore |

*
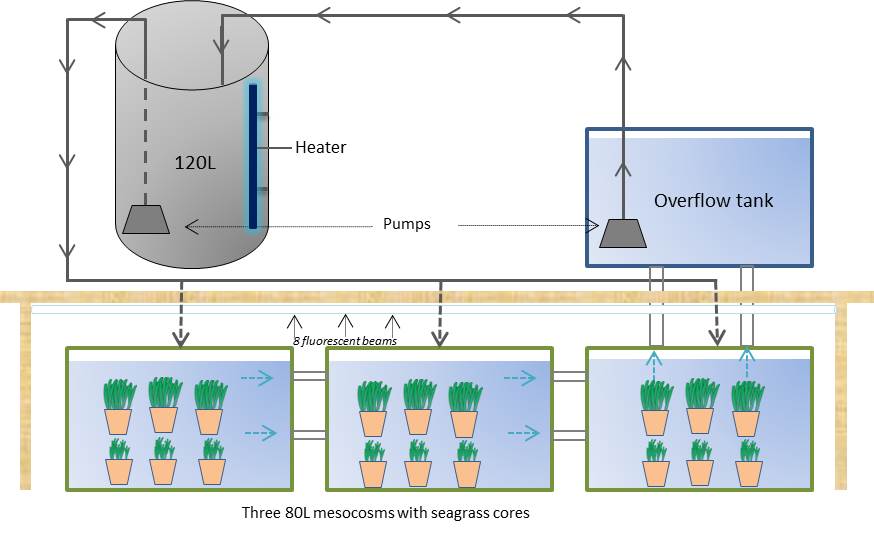
*

**Figure A2:** Infographic of the experimental setup of one of four mesocosm treatments comprising a 120L reservoir with aerated seawater heated to treatment temperature levels (18, 22, 26 and 30^o^C). Water was pumped through an equal release flow through system into three 80L tanks each containing six seagrass replicates - three small-leaved and three large-leaved morphotypes.

**80L**

**80L**
